# Kali Bichromicum

**Published:** 1888-12

**Authors:** W. P. Wesselhœft

**Affiliations:** Boston


### KALI BICHROMICUM.

W. P. Wesselhœft, M. D., Boston.

Mrs. P., aged fifty-five, a fat, light-haired, phlegmatic woman, had frequent nose-bleeds from right nostril, profuse and growing steadily more copious during the past two and a half years. About three years ago she applied a "strong lotion" to her piles, which she thought were weakening her by bleeding. The piles gradually disappeared, and the nose-bleeds commenced at first slightly, but steadily increasing.

In December, 1881, she took one dose of Kali-bich.$^{cm}$. A month later she reported the nose-bleeds were very much less in quantity and frequency, but that several bunches had appeared at once, not bleeding. Five months later she reported frequent trouble with blind piles, which have to be reduced after stool, and sometimes protrude from exertion. Has had no return of nose-bleed for four months. I heard from her the other day ; the report remaining the same.

